# Cardiac Adipose Tissue Segmentation via Image-Level Annotations

**DOI:** 10.1109/JBHI.2023.3263838

**Published:** 2023-06-06

**Authors:** Ziyi Huang, Yu Gan, Theresa Lye, Yanchen Liu, Haofeng Zhang, Andrew Laine, Elsa Angelini, Christine Hendon

**Affiliations:** Department of Electrical Engineering, Columbia University, New York, NY 10027 USA; Department of Biomedical Engineering, Stevens Institute of Technology, Hoboken, NJ 07030 USA; Department of Electrical Engineering, Columbia University, New York, NY 10027 USA; Department of Electrical Engineering, Columbia University, New York, NY 10027 USA; Department of Industrial Engineering and Operations Research, Columbia University, New York, NY 10027 USA; Department of Biomedical Engineering, Columbia University, New York, NY 10027 USA; Department of Biomedical Engineering, Columbia University, New York, NY 10027 USA, and with the NIHR Imperial Biomedical Research Centre and ITMAT Data Science Group, Imperial College London, SW7 2BX London, U.K., and also with the Telecom Paris, LTCI, Institut Polytechnique de Paris, 91120 Palaiseau, France; Department of Electrical Engineering, Columbia University, New York, NY 10027 USA

**Keywords:** Optical coherence tomography, cardiac tissue analysis, deep learning, image segmentation, weakly supervised learning

## Abstract

Automatically identifying the structural substrates underlying cardiac abnormalities can potentially provide real-time guidance for interventional procedures. With the knowledge of cardiac tissue substrates, the treatment of complex arrhythmias such as atrial fibrillation and ventricular tachycardia can be further optimized by detecting arrhythmia substrates to target for treatment (i.e., adipose) and identifying critical structures to avoid. Optical coherence tomography (OCT) is a real-time imaging modality that aids in addressing this need. Existing approaches for cardiac image analysis mainly rely on fully supervised learning techniques, which suffer from the drawback of workload on labor-intensive annotation process of pixelwise labeling. To lessen the need for pixel-wise labeling, we develop a two-stage deep learning framework for cardiac adipose tissue segmentation using image-level annotations on OCT images of human cardiac substrates. In particular, we integrate class activation mapping with superpixel segmentation to solve the sparse tissue seed challenge raised in cardiac tissue segmentation. Our study bridges the gap between the demand on automatic tissue analysis and the lack of high-quality pixel-wise annotations. To the best of our knowledge, this is the first study that attempts to address cardiac tissue segmentation on OCT images via weakly supervised learning techniques. Within an in-vitro human cardiac OCT dataset, we demonstrate that our weakly supervised approach on image-level annotations achieves comparable performance as fully supervised methods trained on pixel-wise annotations.

## I. Introduction

CARDIOVASCULAR disease is the leading cause of death in the United States, with atrial fibrillation alone affecting at least 2.3 million people [29]. Treatment of complex arrhythmias such as atrial fibrillation and ventricular tachycardia is through catheter ablation, which directly destroys the cardiac substrates that cause irregular impulse propagation. However, this treatment is sub-optimal, due to the lack of capability to accurately identify optimal ablation targets. With the knowledge of patients’ heart structure, the ablation strategy can be further optimized by avoiding critical structures and identifying arrhythmia substrates, such as areas with increased amounts of adipose tissues. Recent work has shown that an increased amount of adipose tissues within the myocardium is a substrate for cardiac arrhythmias [7], [8], [12], [46].

Optical coherence tomography (OCT) is a non-destructive optical imaging modality that could provide an ideal balance between a penetration depth of 2 mm with a resolution of 4–10 *μ*m [40]. Recent advances have demonstrated the capability of OCT on capturing myocardial structures such as Purkinje network [54], atrial ventricular nodes [20], sinoatrial nodes [5], and myofiber organization [18]. In addition, it can be used to resolve critical tissue substrates of arrhythmias, such as fibrosis and adipose tissues [38]. With the development of OCT-integrated catheters [14], OCT can image the heart wall in real time through percutaneous access [50], which holds promise to aid catheter ablation.

To benefit from the real-time capacity of OCT imaging, analysis of OCT images is expected to be automated for timely decision making. Evaluation of adipose tissue distribution within a human atrial sample requires pixel-wise analysis of large volumetric datasets [15]. Manually annotating adipose tissues within a single OCT volumes can take a well-trained annotator over 10 hours. Therefore, automated identification of cardiac tissues, especially adipose tissue, in OCT images is greatly needed.

Current automated analysis on cardiac OCT images is mostly based on fully supervised learning models [22], [23], [34]. These models were limited and suffered from the drawback of manual workload in the labeling process. To avoid overfitting, a large amount of data is required to support the model training. For segmentation tasks, the labeling process is extremely time-consuming and has limited accuracy. Moreover, OCT images are volumetric, adding an additional challenge to labeling. Thus, automatic analysis with weakly supervised learning models is of great interest.

Although recent studies have conducted retinal OCT analysis [51], [55], transferring OCT retinal segmentation to cardiac solution for weakly supervised cardiac OCT segmentation is still elusive for three reasons. First, cardiac adipose and fibrosis tissues can appear in multiple sub-regions with irregular shapes and infiltrating patterns. Thus, cardiac OCT images are more complicated than retinal substrates with rather regular layered structures. Second, boundaries between cardiac substrates are more blurry than between the retinal layers. Third, cardiac substrates have a larger variance among patients than retinal tissues.

In this study, we present a weakly supervised learning framework for cardiac tissue segmentation using image-level labels. Our training approach has two stages, namely pseudo label generation and segmentation network training. We first use the class activation map (CAM) results obtained from a binary classification network to generate adipose location seeds. Then, we develop a superpixel-based segmentation algorithm to generate pseudo labels followed by segmentation training. Our contributions are as follows:

1. We propose a weakly supervised learning framework for cardiac tissue segmentation. Our model is trained without the need of pre-training or domain adaptive learning.
2. We combine CAM with superpixel segmentation to effectively address the tissue segmentation challenges caused by irregular shape and blurry boundary in cardiac OCT images.
3. We evaluate our approach on a human cardiac dataset and demonstrate that our weakly supervised model achieves comparable performance with fully supervised algorithms.

## II. Related Work

Regarding tissue analysis on cardiac OCT images, [16] imaged and analyzed features on dense collagen, loose collagen, fibrotic myocardium, normal myocardium, and adipose tissue for automatic classification. In [42], segmentation was obtained from the variance map through compressive sensing reconstruction. In [38], the distributions of adipose tissues and fiber orientations were retracted and mapped throughout human left atrium, while in [6], the visualization of cardiac fibers in the atrium, ventricle, atrioventricular node, and sinoatrial node were presented. Overall, conventional cardiac OCT image analysis relies on handcrafted features for tissue characterization or fiber orientation-based methods to focus on myofibers.

Superpixel is a classic unsupervised segmentation method that has been widely used in biomedical data analysis. Without requirement on pre-annotated training sets, the superpixel based methods group the pixels into homogeneous clusters according to the similarity among pixels. [53] combined superpixel with LogitBoost adaptive boosting to detect glaucomatous damage in 3D OCT images. In [44], the superpixel technique was applied to generate the flexible kernels of local statistics on the Jones matrix-based polarization sensitive OCT.

Deep learning approaches have achieved great success in OCT image segmentation tasks [19], [25], [32], [36], [41], [43], [47]. [47] developed a fully convolutional network with Gaussian process based post processing for retinal OCT segmentation. [13] proposed a novel framework that combined a hybrid convolutional neural network and graph search method for retinal layer boundary detection. [4] developed a fully convolutional network-based AV-Net for artery-vein classification. Their model contained a multi-modal training process that involved both en-face OCT and optical coherence tomography angiography (OCTA) to provide the intensity and geometric profiles. [23] trained a fully supervised segmentation network for cardiac tissue segmentation and used model uncertainty to estimate tissue heterogeneity. Existing work mainly relies upon fully supervised learning techniques.

In contrast to fully supervised methods, weakly supervised approaches use higher level labels including scribbles [26], [35], bounding boxes [28], [52], and image-level labels [51] to guide the pixel-level segmentation training process. [51] successfully segmented lesions by calculating the differences between the input abnormal images and normal-like retinal OCT images from a CycleGAN model. [55] employed a few shot learning technique for retinal disease classification and applied a GAN to enrich normal OCT images with OCT images of rare diseases. [27] proposed a Noise2Noise [33] based weakly supervised learning model for OCTA image reconstruction task. The image-level label is the most convenient and easiest supervision among all weak annotations, as it does not require any detailed annotations in the input images. As a result, it cannot be directly used for segmentation guidance, due to the lack of location clues for the target tissues. By generating the initial location seeds, CAM [57] provides a practical solution to solve this issue, and thus has been widely adopted as the first step of weakly supervised learning frameworks. [10], [21], [56]. [56] developed an end-to-end approach named reliable region mining for weakly supervised semantic segmentation. Combined with CAM, their model applied additional conditional random field operation to get reliable object regions. In [10], a boundary exploration based segmentation approach was proposed to explore object boundaries in the segmentation training process. Researchers in [21] deployed an iterative learning framework to gradually expand the seeded regions.

## III. Problem Analysis

Our study is conducted on a cardiac dataset that was acquired from 44 human hearts with a median age of 62 years. The dataset contains both healthy hearts, end-stage heart failure, atrial fibrillation, coronary heart disease, cardiomyopathy, and myocardial infarction. A detailed clinical characteristic is presented in Section V-A. These various disease conditions might alter the visual features of cardiac substrates, raising the following unique challenges on the algorithm design:

### In-depth image and focal plane.

OCT provides in-depth cross-sectional images in which the x-axis and y-axis are not interchangeable. The signal intensity decreases with increasing axial (depth) distance due to light attenuation as it travels in the tissue. In addition to the depth, the signal intensity is also affected by the system optics and configuration. The optical focus, corresponding to one depth (horizontal row of pixels), will have a higher intensity than regions deeper within the image. As a result, noise distribution within a 2D image is not uniformly distributed.

### Various features and irregular shapes.

**Fig. 1.**
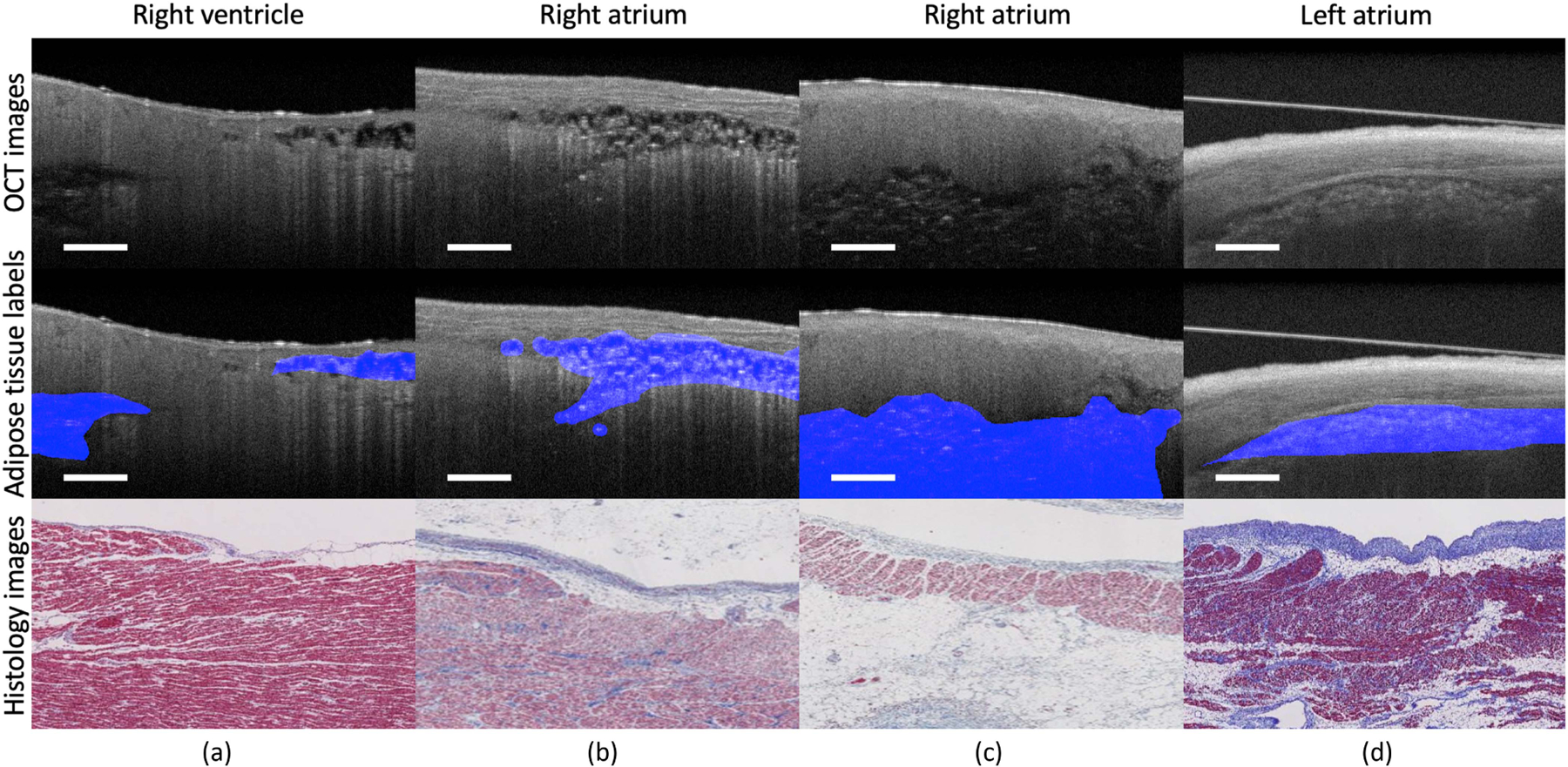
Representative OCT images from cardiac dataset. Sample (a) is obtained from right ventricle. Sample (b) and sample (c) are obtained from right atrium. Sample (d) is obtained from left atrium submerged in PBS solution. The features of adipose tissue present great variations among different locations and imaging conditions. The unclear boundary and irregular shape of adipose tissues add unique challenges for automated segmentation. Scale bar: 500 *µ*m.

As shown in Fig. 1, adipose regions present great variations among cardiac OCT images from human donors with cardiovascular disease due to heterogeneous heart remodeling. In Fig. 1(a), intra-scan inconsistency can be clearly observed in the two sub-regions. Meanwhile, in comparison with Fig. 1(b), the size of fat cells in Fig. 1(a) is much smaller and the number of fat cells is larger, as indicated in the histology images. In addition, the distance to the endocardium tissue can also affect tissue appearance. Adipose tissues in Fig. 1(c) are deeper in the myocardium and appear darker and blurrier than in Fig. 1(b). Finally, the features and shapes can be further impacted by experimental conditions. In Fig. 1(d), the OCT image was obtained from tissues submerged in phosphate buffered saline (PBS). In this sample, the adipose tissues have very low contrast with the surrounding normal tissues. Thus, algorithms relying on shape constraints or boundary exploration cannot be easily extended to this task.

### Similar pattern among adipose tissue and noise.

Image noise and artifacts are inevitable during the acquisition process. Features of adipose tissue are very similar to those of speckle noise and artifacts. To avoid over-smoothing the adipose regions, we do not deploy any image registration/denoising algorithm.

### Data imbalance and limited training data.

In human cardiac samples, the majority of regions are normal tissues, such as myocardium and endocardium, rather than targeted adipose tissues. In our dataset, only around 11% OCT images show visible clusters of adipose tissues. At the pixel level, pixels belonging to adipose tissues only account for 2.6% of the total pixels to label. Even for images that contain adipose tissues, the ratio of number of adipose-related pixels over the total number of pixels is very small. Hence, the samples that are informative for model training are very limited.

### Mathematical modeling:

Let $X=\left\{X_{i};\;\;\;i=1,\ldots ,N\right\}$ denote the set of images. For each image $X_{i}\in X=\left(i=1,\ldots ,N\right)$, the image-level annotation $y_{i}\in \left\{0,1\right\}$ indicates whether or not $X_{i}$ contains the adipose tissues. The training data is denoted by $D_{tr}=\left\{\left(X_{i},y_{i}\right);\;\;\;i=1,\ldots ,N\prime \right\}$, which consists of N′ images $X_{1},\ldots ,X_{N\prime }$ with corresponding image-level annotations $y_{1},\ldots ,y_{N\prime }$. Let $X\prime =\left\{X_{i};\;\;\;i=1,\ldots ,N\prime \right\}$. The goal of our approach is to build a segmentation network $f_{seg}\left(X;\theta \right)$ with network parameters θ, so that it can generate the pixel-wise segmentation masks Pred, which has binary label $Pred\left(\omega \right)$ at each pixel position ω in X.

## IV. Methodology

**Fig. 2.**
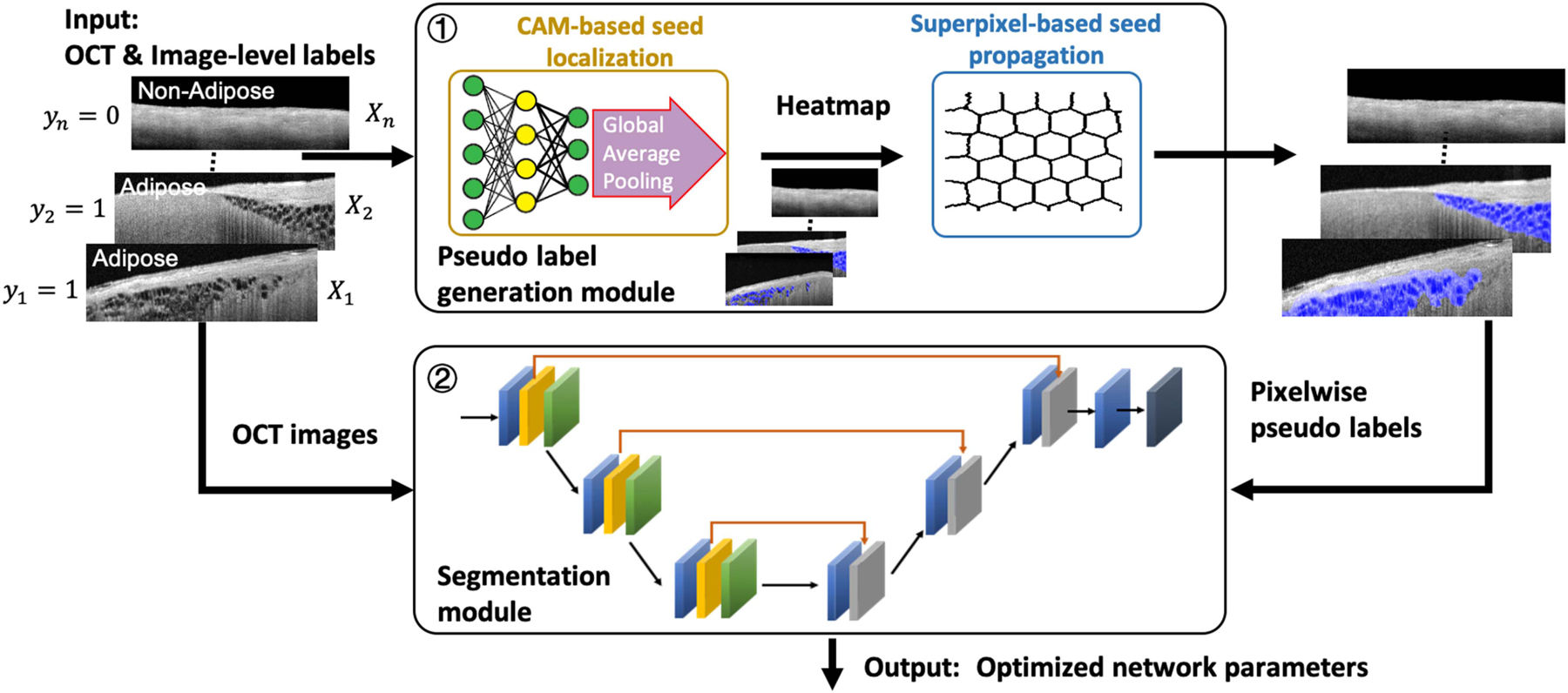
Algorithm training flow of the proposed weakly supervised segmentation approach. The framework consists of two separate modules, namely pseudo label generation and segmentation network training. In pseudo label generation module, pixel-wise pseudo annotations are generated by the integration of CAM and superpixel methods. In segmentation network training module, a segmentation network is trained on the pseudo labels with a novel loss function.

In this paper, we propose a weakly supervised learning framework for cardiac tissue segmentation tasks. Fig. 2 shows the pipeline of our proposed framework. As shown, our training approach consists of two major stages: pseudo label generation and segmentation network training. The pseudo label generation module is formed by two components: we first apply the CAM approach to generate initial adipose seeds and then we use superpixel-base segmentation method to propagate the adipose seeds into pseudo pixel-wise labels. A detailed pseudo algorithm for the pseudo label generation module is listed in Algorithm 1. In the segmentation module, we introduce a novel loss function with a special focus on the adipose seed regions to increase the detection performance of our segmentation network. The proposed method addresses the issue of great variation among adipose tissues for two reasons. First, the proposed method relaxes a predicting precise irregular boundary issue to a rough localization of adipose seed issue using CAM. Without the need of prior knowledge, the CAM can be used to indicate the location of potential adipose tissues. Second, we determine the actual boundary of detected adipose using an unsupervised way, superpixel segmentation, thus bypassing the challenge of learning from large amounts of adipose regions with great boundary variation.

### A. Pseudo Label Generation

#### 1) CAM-Based Seed Localization:

The first stage of our model is to find reliable adipose seeds to indicate the location of adipose tissues. We follow the learning steps in [57] to get the initial CAM to indicate the location of adipose regions. To identify the extent of target tissue regions, we employ the global average pooling (GAP) layer to the last convolution layer of the classification network. The final prediction of the network is classified by a fully connected layer. After model training, the CAM results for the adipose tissues are obtained as follows:

$$M_{adipose}\left(\omega \right)=\sum_{j}\mu_{j}f_{j}\left(\omega \right)$$

where $f_{j}\left(\omega \right)$ is the activation of unit j in the last convolutional layer at spatial location ω and $\mu_{j}$ is the weight corresponding to the adipose tissue for unit j.

The class activation maps have strong responses on regions with artifacts and high-intensity noise. To increase the reliability of pseudo label generation, we apply a boundary masking algorithm on the class activation maps to filter out adipose seeds that are located in the background regions (false positive caused by noise) and regions close to the tissue-background boundary (false positive caused by artifacts). We adopt the cardiac layer segmentation algorithm from [16] and use the boundary of the top generated layer as the tissue-background interface. After getting the tissue surface, we remove adipose seeds above or close to the tissue-background boundary.

#### 2) Superpixel-Based Seed Propagation:

Superpixels are generated as in [2] with an approximate number of superpixels as 2000. An entire superpixel is labeled as adipose tissue if one of its inner pixels is labeled as adipose tissue. Upon the generation of superpixels, the initial segmentation pseudo labels can be further improved to eliminate the following two misclassifications: 1) The adipose seeds may omit some adipose regions, and 2) the adipose seeds may incorrectly mark the normal regions as the adipose regions, due to the artifacts and intensity noise. To further remove the noisy annotations, we apply the Markov spatial regularization strategy [19] to add the ignored regions and remove the noisy adipose superpixels which only contain the normal tissues. Since the adipose cells are clustered in the cardiac tissue, the neighbors of an adipose region are more likely to belong to the adipose tissue clan, while small isolated adipose superpixels are more likely to be the normal regions corrupted by noise. Based on these criteria, we develop a simple yet effective spatial regularisation strategy: the label of a superpixel will be updated if most of its neighbors (≥ 80%) belong to another class.


$$\underline{\begin{array}{l} \bar{\underline{\text{Algorithm}\;1:\text{Algorithm}\;\text{Framework}\;\text{for}\;\text{Pseudo}\;\text{Label}\;\text{Generation}\;\text{Module}.}}\\ \;\text{Input}:\text{Training}\;\text{dataset}\;X=\left\{X_{1},X_{2},\ldots ,X_{n}\right\}\;\text{with}\\ \;\text{image}-\text{level}\;\text{labels}\;Y=\left\{y_{1},y_{2},\ldots ,y_{n}\right\};\\ \;\text{Output}:\text{Pixel}-\text{wises}\;\text{pseudo}\;\text{labels}.\\ \;\text{Procedure}:\\ \;\text{Step}\;1:\text{Train}\;\text{localization}\;\text{network}\;\text{from}\;X\;\text{and}\;Y.\\ \;\text{Step}\;2:\text{Apply}\;\text{the}\;\text{CAM}\;\text{method}\;\text{to}\;\text{generate}\;\text{the}\;\text{initial}\\ \;\text{tissue}\;\text{seed}\;\text{results}\;C=\left\{c_{1},c_{2},\ldots ,c_{n}\right\}.\\ \;\text{Step}\;3:\text{Apply}\;\text{the}\;\text{boundary}\;\text{masking}\;\text{on}\;C\;\text{and}\;\text{get}\\ \;\text{updated}\;\text{tissue}\;\text{seeds}\;\hat{C}.\\ \;\text{Step}\;4:\text{Apply}\;\text{superpixel}-\text{based}\;\text{propagation}\;\text{method}\;\text{on}\;\hat{C}\\ \;\text{to}\;\text{generate}\;\text{the}\;\text{initial}\;\text{pseudo}\;\text{segmentation}\;\text{labels}\;S.\\ \;\text{Step}\;5:\text{Update}\;S\;\text{with}\;\text{the}\;\text{spatial}\;\text{regularisation}\;\text{strategy}\\ \;\text{and}\;\text{get}\;\text{the}\;\text{final}\;\text{pseudo}\;\text{segmentation}\;\text{labels}\;\hat{S}. \end{array}}$$

### B. Segmentation Network Training

Adipose tissues are sparse compared with normal tissues, such as myocardium and endocardium. Without special consideration, the segmentation performance might be severely limited due to data imbalance. To overcome this challenge, we use an adipose seed loss, inspired by [31], to optimize our segmentation network. In comparison with the original form of seed loss in [31], our adipose seed loss function only focuses on the regions of target tissues while the background regions are omitted. The final outputs of our segmentation network are pixel-wise segmented adipose location results.

We first introduce the loss function. Let $p_{k}\left(\omega \right)$ denote the network output probability for class k at the pixel position ω∈Ω with $\text{Ω}\subset {\mathbb{R}}^{2}$ and $s_{k}\left(\omega \right)$ denote the pseudo-pixel-wise label where $k\in \left\{0,1\right\}$. The cross-entropy loss (CEL) and adipose seed loss (ASL) is defined as follows:

(1) $$CEL=-\frac{1}{\left|\Omega \right|}\sum_{\omega \in \Omega }\sum_{k=0}^{1}s_{k}\left(\omega \right)\text{log}\left(p_{k}\left(\omega \right)\right)$$

(2) $$ASL=-\frac{1}{\left|\Omega_{1}\right|}\sum_{\omega \in \Omega_{1}}s_{1}\left(\omega \right)\text{log}\left(p_{1}\left(\omega \right)\right)$$

where ${\text{Ω}}_{1}=\left\{\omega \in \text{Ω}:s_{1}\left(\omega \right)=1\right\}$ is the set of spatial locations that are pseudo-labeled with class 1 (i.e., the adipose class). Compared with the CEL (1), the ASL (2) only focuses on the regions of adipose tissue, and thus, it helps to reduce the impact of the false negatives in the pseudo segmentation labels.

We also use Dice loss (DL) in our loss function to learn the context information. The DL is defined as:

(3) $$DL=1-\frac{1}{2}\sum_{k=0}^{1}\frac{2\sum_{\omega \in \text{Ω}}\left(p_{k}\left(\omega \right)s_{k}\left(\omega \right)\right)}{{\sum_{\omega \in \text{Ω}}\left(p_{k}\left(\omega \right)\right)}^{2}+\sum_{\omega \in \text{Ω}}{\left(s_{k}\left(\omega \right)\right)}^{2}}$$

Finally, our segmentation network $f_{seg}\left(X;\theta \right)$ is jointly optimized by the combination of CEL, ASL, and DL:

(4) $$Loss=\alpha_{1}\cdot CEL+\left(1-\alpha_{1}\right)\cdot ASL+\alpha_{2}DL$$

where $\alpha_{1}$ and $\alpha_{2}$ are weight hyper-parameters. The sum of CEL and ASL is equivalent to a weighted loss. For better understanding, we use the form in (4) to separate the weight of CEL and ASL.

## V. Experiment Evaluation

### A. Dataset

We evaluate the performance of our proposed model on the human cardiac dataset previously used in [15]. It consists of *in-vitro* cohort of 385 images taken from 44 human atria and ventricles using the Thorlabs OCT system. The samples were acquired through a National Disease Research Interchange approved protocol from Columbia University. All specimens were de-identified and considered not human subjects research, according to the Columbia University Institutional Review Board under 45 CFR 46. Table I presents the clinical characteristic of the human donor hearts. A detailed feature analysis study among different group of people was presented in [39]. The source of the Thorlabs OCT system was centered at 1325 nm with a bandwidth of 150 nm. The axial and lateral resolutions were 6.5 and 15 *μ*m in air, respectively. All datasets were acquired at 28 kHz. Each OCT image is of size 512 × 800 pixels with a field of view of 2.51 mm × 4 mm. We crop the images into small overlapping patches with size of 512 × 128 pixels. Three experts, blinded to the algorithm design, annotate the OCT images under the guidance from a pathologist. All images are carefully annotated at pixel level with visual cron-check on corresponding histology images. Our evaluation is conducted on a five-fold cross validation strategy with validation sets randomly divided over human

### B. Implementation Details

#### Seed localization network.

To avoid overfitting, we only train a localization network with three hidden layers for adipose tissue seed generation. We use the ReLU function as the activation function. The number of channels in each hidden layer is 32, 32, and 64. We applied the GAP layer to the last convolutional layer to learn the cluster pattern of adipose tissues. The output of the GAP layer is classified with a fully connected layer. The digital resolution of output CAM drops down to $\frac{1}{4}$ of original digital resolution after passing through the localization network. The networks are optimized on cross entropy loss via Adam optimizer [30] with random Glorot uniform initialization [17]. The batch size used for training is 16. Over the cross validation sets, all networks converged within 300 epochs with a learning rate of $1e^{-3}$. After normalizing, the background threshold in CAM generation is 0.15.

#### Segmentation network.

We employ the classic medical segmentation network UNet [48] as the baseline of our learning framework. In the loss function (4), the hyper-parameter $\alpha_{2}$ is 0.5 while $\alpha_{1}\in \left[0,1\right]$ is determined by the proportion of adipose tissues in the training set: $\alpha_{1}=1$ if the ratio of adipose images is less than 5%. Otherwise, $\alpha_{1}=0.5$. All segmentation networks were randomly initialized and converged within 200 epochs with a learning rate of $0.1^{-3}$, batch size 32, and weight decay $10^{-4}$. Our study is based on the Keras [11] and TensorFlow [1] deep learning frameworks. All experiments are conducted on a computer with the following features: an Intel core i9–9900 K (16 M Cache, up to 5.00 GHz) CPU and a RTX 2080 Ti GPU. The number of training images in a cross-validation set is around 300 images, with a size of 512 × 800 pixels. In the preprocessing stage, the training images were cropped into around 1800 overlapping patches (512 × 128 pixels) to enrich the training size. The total execution time for a single trial, which includes the following steps: preprocessing, classification network training, generation of pseudo labels, and segmentation network training, is less than 3 hours.

### C. Evaluation Metrics

In our experiment, we use accuracy and precision to evaluate the overall accuracy and the detection performance of adipose seed results. For pseudo label generation and segmentation evaluation, we use true positive rate (detection rate), false positive rate, and Dice coefficient (F1 score) to evaluate the tissue segmentation performance.

### D. Evaluation of Pseudo Label Generation

#### Adipose tissue seed localization.

**Fig. 3.**
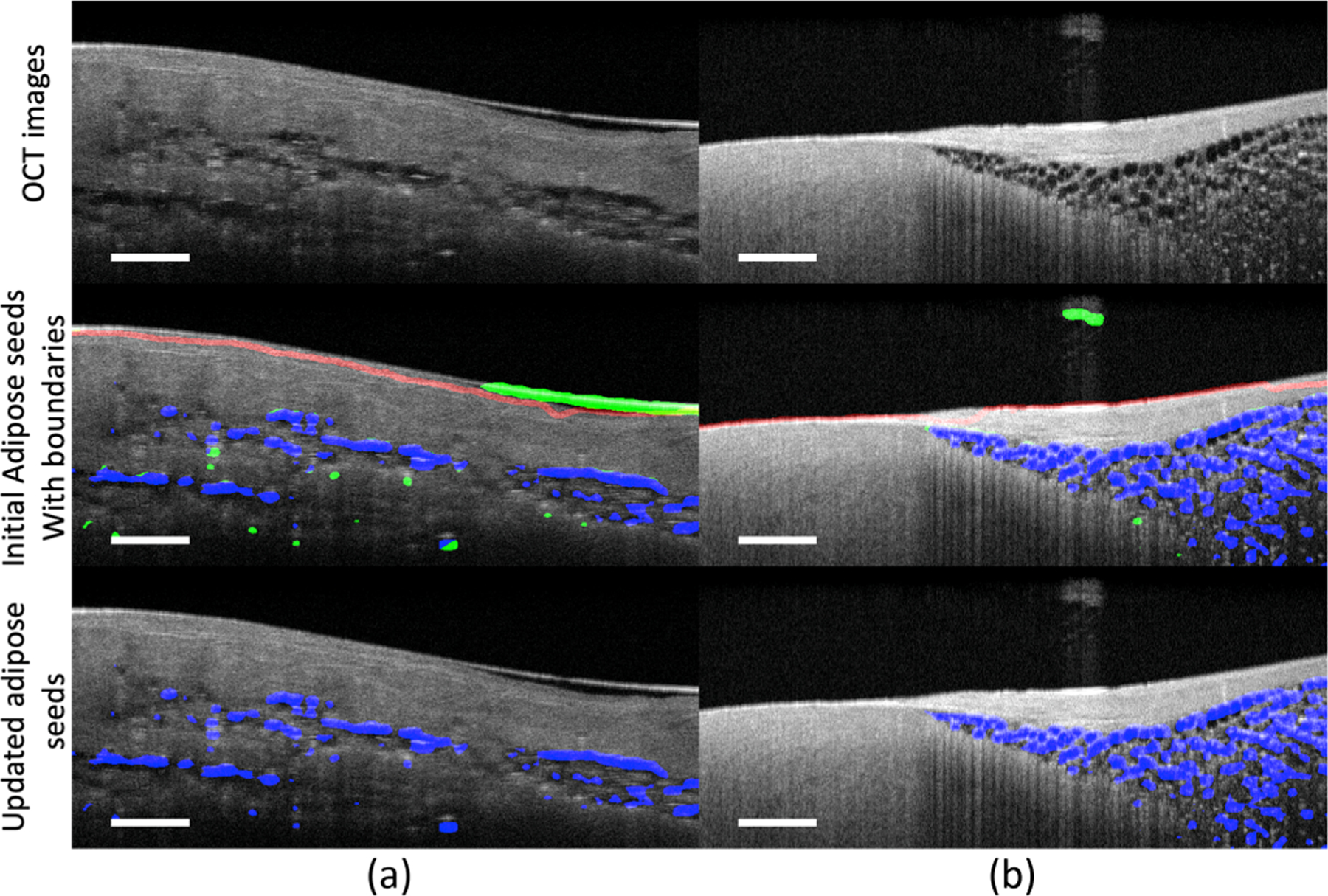
Comparison of tissue seeds before and after the boundary masking algorithm. Red: the detected tissue-background boundaries; blue: accurately annotated adipose seeds; green: false positives. As shown, the boundary masking algorithm can effectively remove the false positive adipose seeds caused by the artifacts and noise. Benefiting from it, the adipose seeds are more precise to be propagated for segmentation guidance. Scale bar: 500 *µ*m. Sample (a) is obtained from the left ventricle. Sample (b) is obtained from the right ventricular septum.

The binary accuracy for our proposed localization network achieves very stable performance on all validation sets. Fig. 3 presents two representative adipose seed results generated from our localization network. In Fig. 3, the detected tissue-background boundary is delineated in red, and the accurately located adipose seeds are marked in blue, with misclassified adipose seeds marked in green. As seen, the boundary masking algorithm can effectively remove the misclassified edges and background noise from the original adipose seed results. Meanwhile, these results also demonstrate the capability of recognizing various types of adipose features using our proposed network. In the lower-left region of Fig. 3(a) and lower region of Fig. 3(b), the adipose region is out-of-focus, and the wall of adipose cells is not distinctive. In the majority of parts of Fig. 3(b), the adipose regions are in focus and appear in the honeycomb structures. These adipose regions are correctly identified using our proposed approach. In Table II, we report the accuracy and precision of adipose seeds before and after boundary masking. As shown, both accuracy and precision are improved after applying the boundary masking algorithm. In particular, the precision of adipose seeds has been significantly increased by approximately 20%, which further demonstrates the effectiveness of boundary masking on accuracy improvement.

**Fig. 4.**
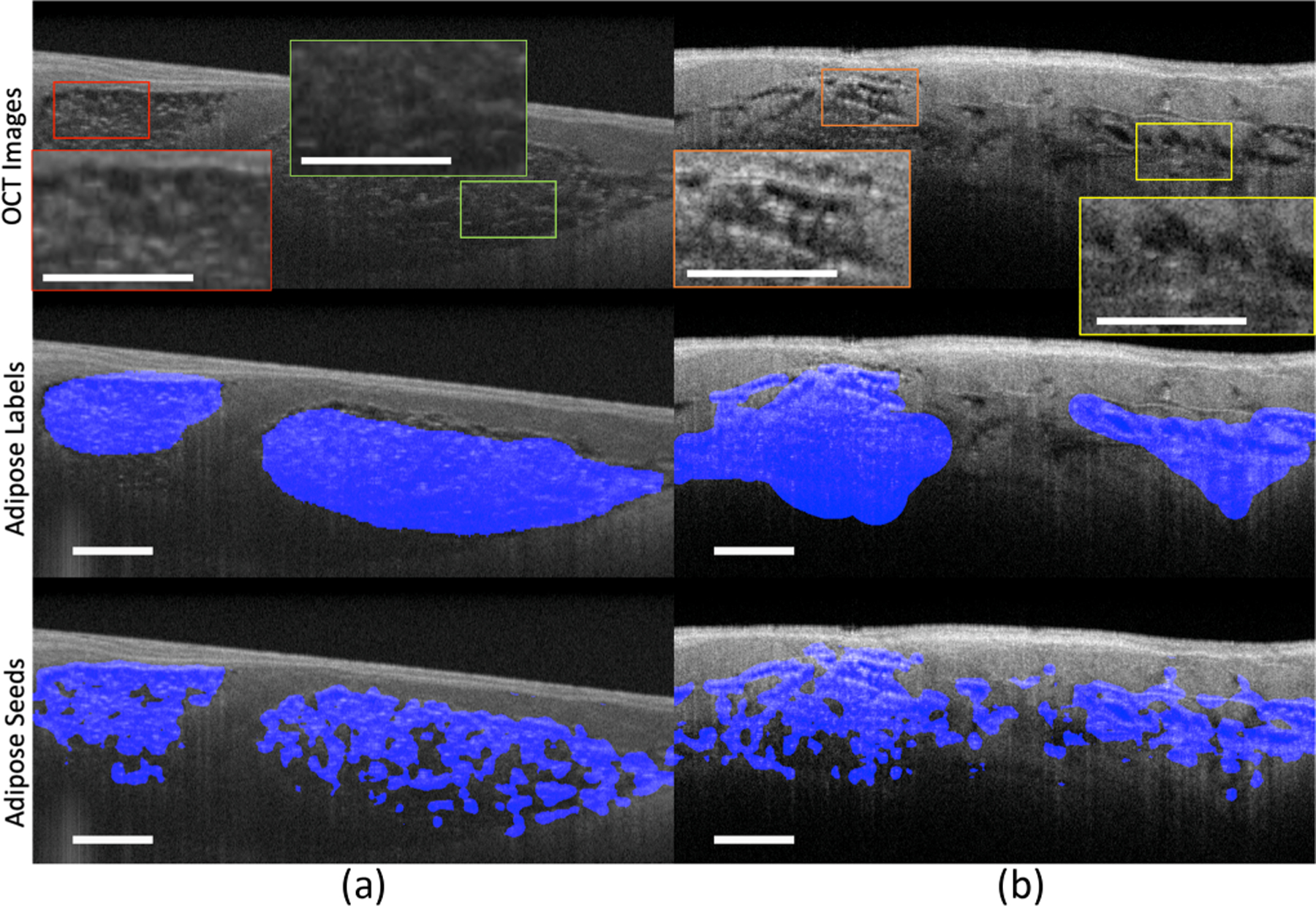
The tissue seed results in samples with different adipose features. Sample (a) is obtained from the left atrium. The highlighted regions in sample (a) are two regions with different focal statuses. The red box is within the focal plane while the green box is out of focus. Sample (b) is obtained from the right ventricle. The highlighted regions in sample (b) are with different tissue structures. The orange box is with multiple small fat cells while the yellow box is with a few big fat cells. Scale bar: 500 *µ*m.

In Fig. 4, we show two representative samples that were obtained from different chambers. In each sample, the adipose tissues are located in multiple regions with different appearances. As shown, our model can successfully localize the adipose tissue in a single sample with different adipose appearances. These results indicate that our model is able to solve the various feature challenges described in Section III.

#### Pseudo label generation.

**Fig. 5.**
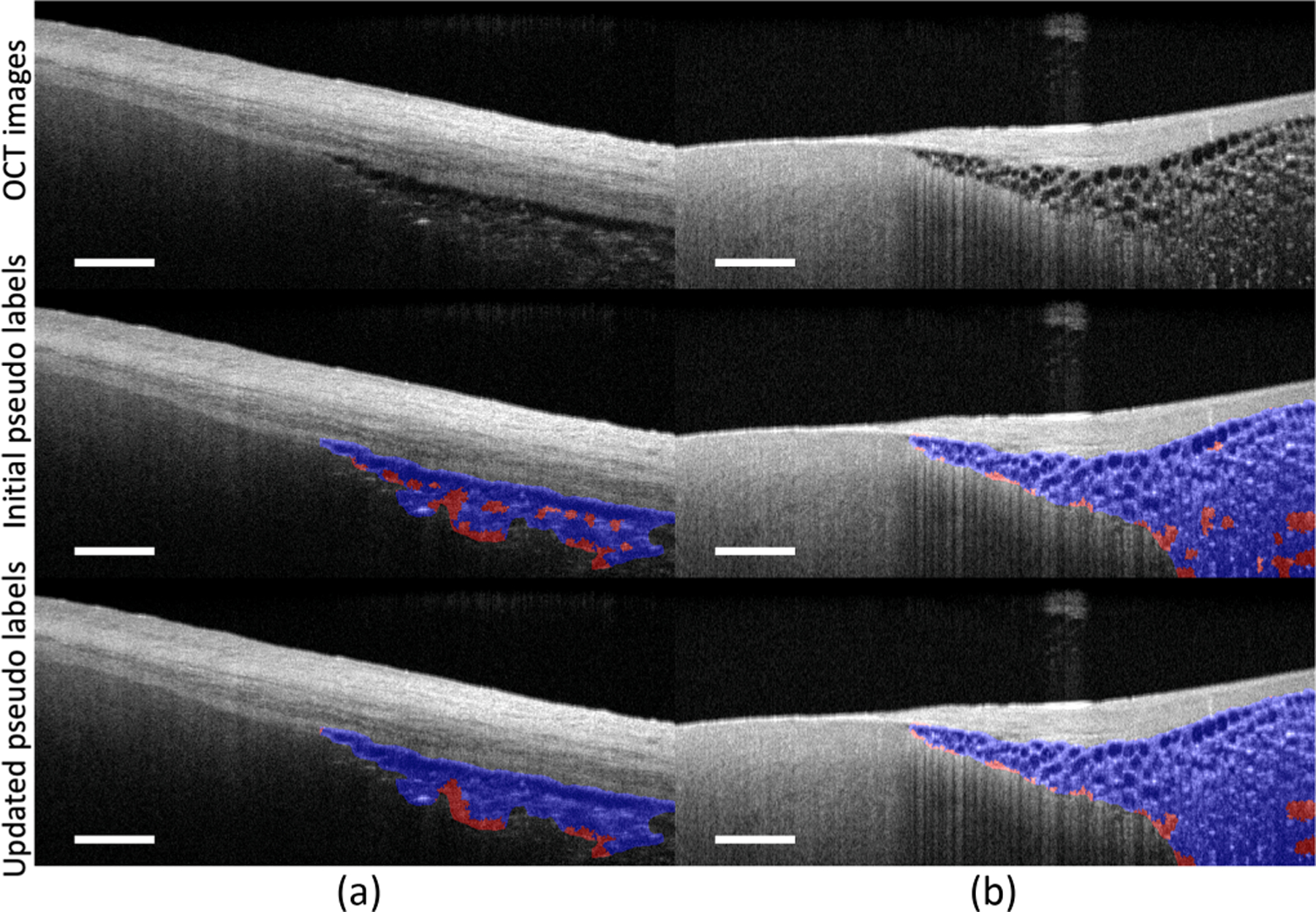
Comparison of pseudo labels with and without the spatial regularisation strategy. Blue: accurately annotated adipose pixels; red: false negatives. The spatial regularisation strategy helps to correct the mis-labeled pseudo labels by using the context information from nearby regions. After applying it, the false negatives have been significantly reduced. Scale bar: 500 *µ*m. Sample (a) is obtained from the left atrium. Sample (b) is obtained from the right ventricular septum.

Fig. 5 shows two superpixel segmentation results with accurately segmented pixels marked in blue and false negatives marked in red. In Table III, we provide a quantitative evaluation of our spatial regularisation strategy on samples with adipose tissues. There is an increase of 2% in Dice coefficient after applying it.

#### Localization network architecture.

To avoid overfitting, we apply a small localization network with 32, 32, and 64 channels on CAM generation. To learn the impact of network architecture on pseudo label generation, we conduct sensitivity analysis on models with more parameters: a deeper network with 32, 32, 32, and 64 channels and a wider network with 64, 64, and 64 channels in corresponding convolutional layers. Table IV presents the evaluation metrics on pseudo labels generated from different networks over the adipose samples. The quality of pseudo labels from deeper and wider networks is slightly worse than our proposed network. This discrepancy is probably caused by the noise and artifacts in the OCT images, as these large models might overfit to some non-robust features [24].

#### Sensitivity analysis on the number of superpixels.

**Fig. 6.**
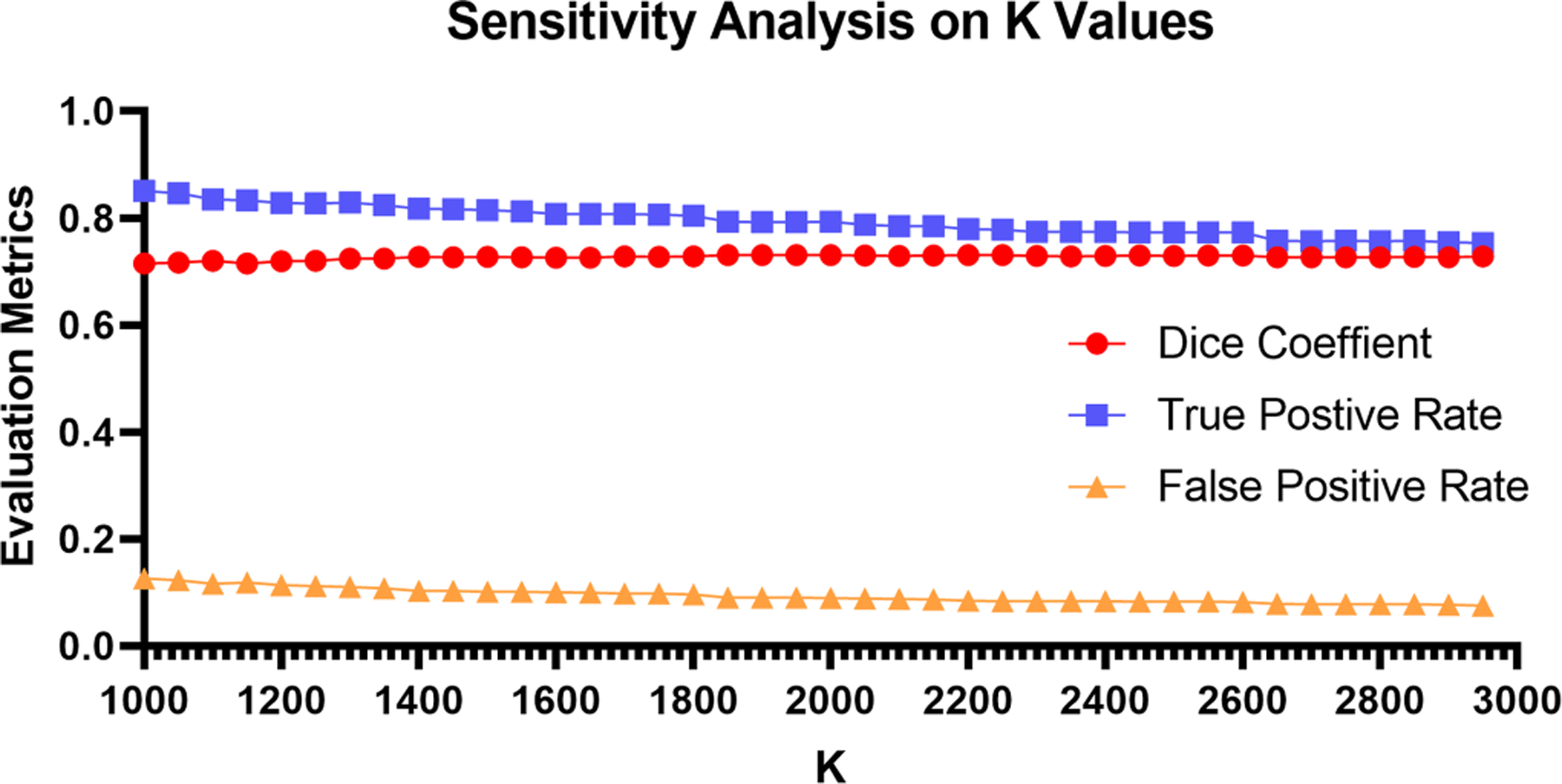
Impact of the number of superpixels on pseudo label quality. The quality of pseudo labels only has slight variance on the evaluation metrics (Dice coefficient, true positive rate, and false positive rate) among different K values, which indicates the robustness of our model on pseudo label generation.

The number of superpixels K is the most important hyperparameter in the pseudo label generation module, with larger K values leading to smaller sizes of superpixels. To learn the impact of K on pseudo label quality, we report the evaluation metrics of pseudo labels with different K values in Fig. 6. Along with increased K values, the true positive rate slightly decreases while the false positive rate is improved. Thus, we empirically set K=2000 to balance the true positive rate and false positive rate in our study.

### E. Evaluation of Segmentation Performance

#### 1) Cross Validation Experiments:

We use fully supervised models trained from pixel-wise accurate segmentation masks as the baseline for comparison. Table V summarizes the averaged results and the standard deviation of our proposed weakly supervised approach and fully supervised baselines. To fully evaluate our model, we report the results calculated from all samples.

##### Weakly supervised learning vs fully supervised learning.

Our weakly supervised model, trained from image-level labels, achieves comparable quality results to the fully supervised model that trained on pixel-wise labels. In addition, our dataset was acquired within a time frame that spanned over five years. During this time frame, imaging setup, such as sample freshness, imaging condition, and tissue preparation, varied among experiments. Thus, our results also demonstrate the generalization ability of our model against imaging condition variance, showing its strong potential on real-world clinical applications.

##### Comparison with different segmentation models.

In addition to the classic UNet model, we also evaluate the performance of our approach with other state-of-the-art segmentation models including DeepLab V3 [9] and FCN [37]. The evaluation metrics of these models are shown in Table V. As shown, all models achieve comparable results (true positive rate *>* 0.80, false positive rate *<* 1.5 over all samples), which indicates the genericity of our framework on weakly supervised tissue identification.

##### Comparison with existing weakly supervised learning framework.

We compared our method with two existing weakly supervised learning frameworks: reliable region mining (RRM) [56] and CycleGAN [51]. As shown in Table V, our methods generally produce a higher true positive rate and a lower false positive rate than RRM and cycleGAN, independent of segmentation models (i.e., U-Net, FCN, and DeepLab). RRM is based on the concept of conditional random field (CRF). CRF was originally designed for natural images where the boundary of objects was with high contrast, and pixel-wised affinity could be calculated accordingly. However, the blurry boundary of adipose regions within OCT images can add additional challenges in tissue segmentation and thus lead to lower performance of RRM. In contrast, the combination of superpixel design and Markov regularization can contribute to addressing the unique challenges that adipose and speckle noise happen to be similar.

Our approach also achieves higher performance than the CycleGAN-based method [51], which relies heavily on spatial constraints. In our problem setting, the adipose tissue could appear in any region under the endocardium. The morphological change is significant compared to those within retinal images [51]. Furthermore, their model detected the bottom layer (RPE) of the retina to avoid the interruption of background noise. However, in the cardiac OCT dataset, there does exist a distinctive bottom layer to avoid background noise.

Although the adipose tissues and the noise are similar on pixel-wise, they are different on cluster-wise. The walls of adipose cells can be differentiated from the background when the adipose regions are close to the endocardial surface (first layer). As the location of adipose regions appears deeper within the tissue, within the OCT image, it is farther from the focus and thus is blurred and has a lower contrast with the background. The superpixel, as a basic unit, carries more regional information than a single pixel, thus showing promise to distinguish the adipose and noise. In addition, the use of Markov spatial regularization could also help to filter isolated noise by removing noise if the majorities are non-adipose regions and expanding regions if the majorities are adipose regions.

##### Ablation study.

Performance drops are observed in Table V when the boundary masking algorithm and spatial regularisation strategy are removed. In particular, after removing the boundary masking algorithm, the true positive rate is severely decreased along with an increased standard deviation. This result indicates the necessity of using a boundary masking algorithm to improve adipose seed quality at the early stage of pseudo-label generation. In comparison with the false positive rate, the true positive rate has notable changes after applying the spatial regularisation, which shows its effectiveness in false negative correction. We further conduct experiments to assess the influence of different loss functions in our proposed model. As shown in the last four rows of Table V, our model which is optimized with adipose seed loss and Dice loss achieves the best true positive rate, while the version without adipose seed loss (CE loss and Dice loss), achieves the best false positive rate in ablation study. The use of seed loss can notably increase the model detection performance but meanwhile, hinder the false positive rate. In contrast, the cross entropy loss is more efficient in controlling the false alarms. These results show that the use of seed loss can efficiently reduce the impact of false negatives in the pseudo labels. This detection rate and false alarm trade-off can be balanced through adjusting the weights of seed loss and cross entropy loss.

#### 2) Representative Segmentation Results:

In this section, we present the visual output of our proposed weakly supervised model in overall performance, small adipose tissue region detection, and 3D segmentation.

##### Overall performance.

**Fig. 7.**
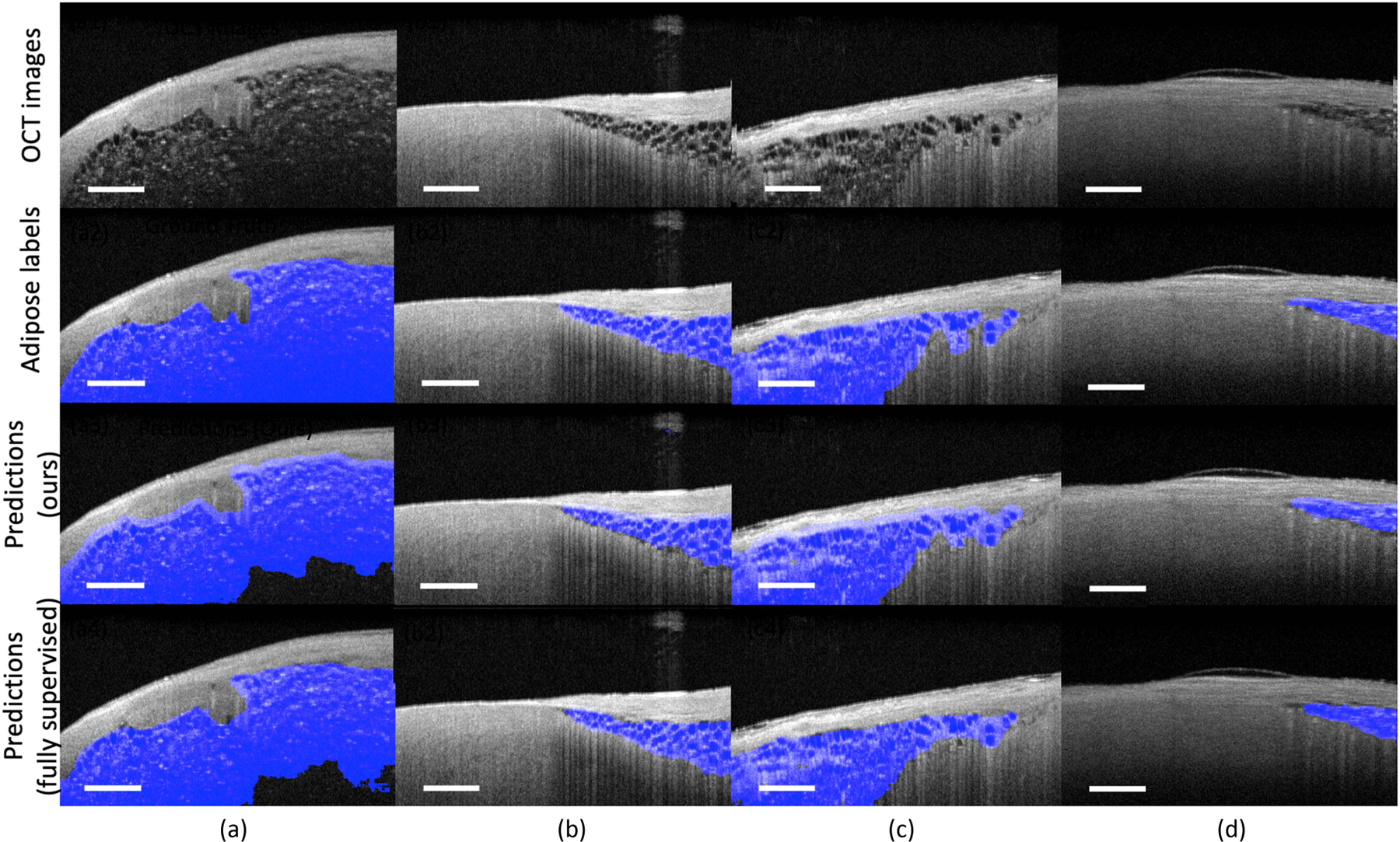
Representative segmentation results from human atrium and ventricle samples. Our proposed approach accurately identifies the adipose tissues located at different regions with various sizes and shapes. All prediction results are highly consistent with the ground truth labels. Scale bar: 500 *µ*m. Sample (a) and sample (b) contain adipose tissue in arbitrary shapes. Sample (c) contains adipose tissue at multiple locations. Sample (d) contains adipose tissue located at regions that are slightly off the focus.

Fig. 7 shows the predicted tissue maps on four human cardiac samples. In Fig. 7(a) and (b), our model accurately localizes the adipose tissue regions in arbitrary shapes. Meanwhile, in Fig. 7(a), we can also observe the over-segmented regions (regions at the left corner) in the ground truth figure. Human annotators tend to over-segment the regions below the penetration depth, while for the network, it may identify these regions as non-adipose tissues because of the low signal-to-noise ratio. This over-segmentation tendency can lead to decreased values in evaluation metrics. In Fig. 7(c), our model successfully identifies the adipose tissues in the multiple regions with different penetration depths. In Fig. 7(d), we show a human atrium sample that is slightly off the focus. Similar to previous results, our models still accurately differentiate the adipose tissues from other tissues, showing their robustness over different image qualities. In all cases, the predicted results are highly consistent with the ground truth labels. These results demonstrate the learning ability of our model via image-level labels, showing its effectiveness in clinical tissue identification.

##### Identifying small adipose tissue regions.

**Fig. 8.**
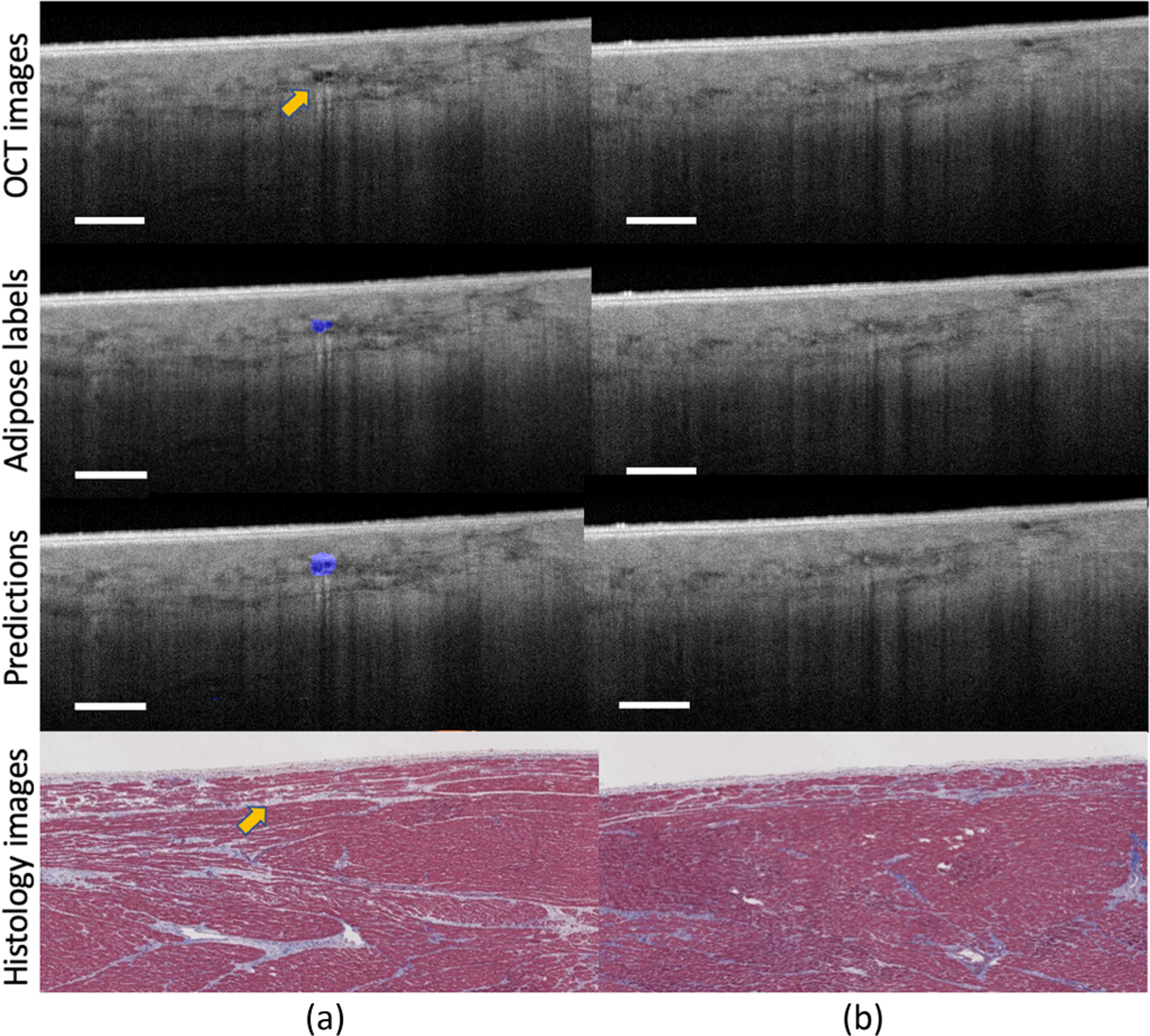
The prediction results of images obtained from nearby regions. Our approach successfully pinpoints the adipose tissues from other tissue types, showing its strong identification ability on adipose tissues. Scale bar: 500 *µ*m. Sample (a) and sample (b) are obtained from nearby regions within the same human heart.

Fig. 8 presents two images obtained from nearby regions within the same human heart. As shown, Fig. 8(a) and (b) are very similar and they all contain large regions of fibrosis tissue. However, in Fig. 8(a), there is a small cluster of adipose tissue surrounded by the fibrosis tissues, while in Fig. 8(b), there is no adipose tissue. As shown, this is a very challenging segmentation task due to the size of adipose tissue and the blurry boundary between different tissue types. Our model accurately delineates the adipose tissue regions in Fig. 8(a) and it does not put any false alarm in Fig. 8(b). In both cases, our model successfully distinguishes adipose tissues from other cardiac substrates. These results further demonstrate the strong learning ability of our model, as it can learn the most discriminative features via image-level labels, rather than simply memorizing the training samples.

##### Visualization of 3D segmentation.

**Fig. 9.**
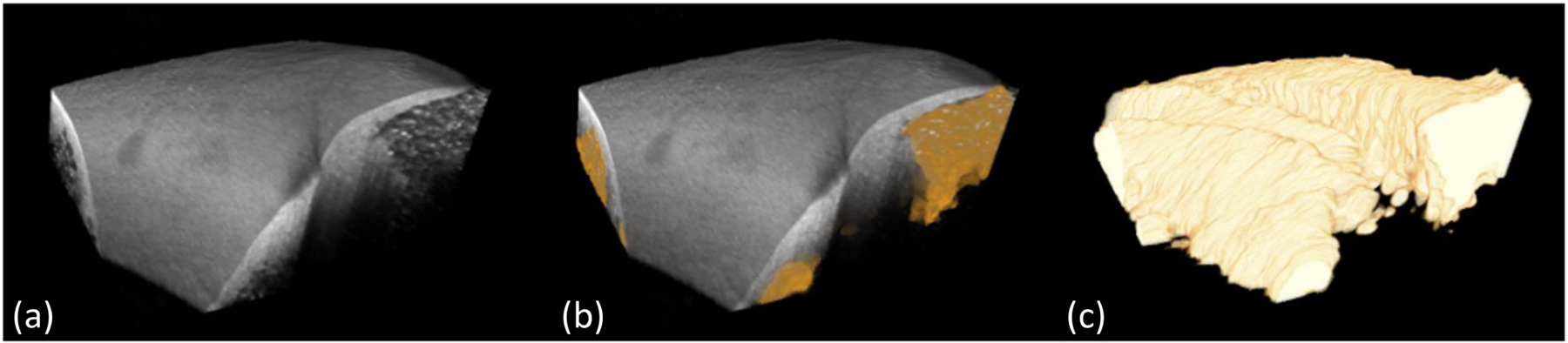
3D visualization of adipose tissue segmentation. (a): the original OCT volume; (b): the original volume overlaid with segmented adipose regions; (c): the segmented adipose regions from proposed approach. The segmented boundaries accurately delineate the morphological changes in adipose shape.

Fig. 9 shows a typical result of 3D visualization of adipose tissue segmentation. We sequentially apply the trained network to segment consecutive Bscans and align segmented Bscans in 3D space. As shown, the segmented boundaries accurately delineate the morphological changes in the adipose tissues. Even though our model is trained on a small quantity of training data with image-level labels, it still successfully segments adipose regions of various sizes. These results indicate that our model has great potential to be applied to assess adipose tissue regions in catheter-based ablation operations.

## VI. Discussion

In this study, we propose a weakly supervised learning framework for cardiac adipose tissue segmentation on OCT images. Our approach contains two powerful modules: the pseudo label generation and the segmentation network training. In the pseudo label generation module, we use the superpixel-based propagation algorithm to address the sparse location seed challenge raised in the CAM results. Benefiting from our boundary masking algorithm and spatial regularisation strategy, the quality of the pseudo labels has been significantly improved for the training guidance. In the segmentation network training module, we introduce a novel loss function to increase adipose tissue detection performance. By evaluating on a human cardiac dataset with cross-validation strategy, our model achieves comparable results with the fully supervised baseline, showing its effectiveness on tissue characterization.

Our study can provide valuable detail on adipose distribution within myocardium regions, holding promise to improve cardiac therapies including ablation procedure and endomyocardial biopsy. Previous research has shown that the accumulation of adipose tissue in ventricular myocardium is associated with severe cardiac arrhythmias such as left ventricular tachycardia and arrhythmogenic right ventricular cardiomyopathy. By pointing out the location of abnormalities, our study could directly improve the radiofrequency ablation treatment, reducing the risk of treatment recurrence and complications. In addition, the knowledge of adipose distribution could also provide referral suggestions on ablation parameters for lesion formation, as the biophysical properties are various among different tissue types. Moreover, our approach also holds promise to be extended for weakly supervised segmentation of adipose tissue in other applications, such as adipose detection in breast cancer classification.

Previous research has shown that the accumulation of adipose tissue in the ventricular myocardium is a substrate for cardiac arrhythmias, which is associated with left ventricular tachycardia and arrhythmogenic right ventricular cardiomyopathy. Additionally, about 75% to 80% of cases of sudden cardiac death are caused by ventricular arrhythmias, and over 80,000 people are detected with supraventricular tachycardia annually [29], [45]. However, there are no existing methods for real-time image guidance of ablation procedures, to the best of our knowledge. Current guidance of ablation procedures is through the measurement of electrograms. Low voltage measurements can be due to increased amounts of collagen (scar or fibrosis) or adipose. Knowledge of the substrate will provide the necessary feedback to determine how to interpret functional measurements. Future OCT-enabled assessment of the presence of adipose (true positive rate) will provide a valuable addition to an electrophysiologist’s toolbox for treating cardiac arrhythmias.

Similar to the experiments in RRM, we notice that other weakly supervised methods that were originally developed in computer vision might not be easily applied to our unique OCT task. For instance, SEC [31] is a CRF-based method. Note that the CRF-based method is originally designed for natural images where the boundary of objects is high contrast and continuous. However, some adipose regions in OCT images do not satisfy this condition, especially when the adipose cells are in out-of-focus regions. Likewise, the pairwise affinity-based IRN [3] approach is also hard to handle such subtle feature variation challenges in OCT adipose images. In addition, the IRN framework relies upon multiple instances of CAM, where each object is considered as a single subject for instance segmentation. Such design cannot be easily integrated with adipose tissue which shows a sparse distribution within the myocardium (shown in Fig. 3), as this sparsity could lead to over hundreds of instances in a single OCT image. Moreover, erasing-based methods [49] could not be applied to our problem setting as normal tissue can be likely removed with fat cells due to the blurry boundary of the adipose region.

One limitation of this study is that the results are evaluated on a benchtop OCT system. To move towards the aid of ablation procedures, a catheter-based OCT system is needed, as it can help to optimize the treatment strategy by providing real-time cardiac substrate information. In the future, we will extend our current work into catheter-based *in-vivo* OCT images. Such extension will require further investigation of challenges such as image quality degeneration and motion disturbance. Compared with benchtop OCT images, catheter-based OCT images are with lower image quality, suffering from lower contrast and motion effects. Without special consideration, the decreased image contrast may hinder the performance of the model. Motion disturbance caused by breath and heartbeat is another important factor that could lead to performance degradation. These disturbances could be partially corrected by applying low-pass filters. In the future, we will also extend our proposed weakly supervised framework to other OCT segmentation tasks, such as breast images and retinal images. Moreover, the segmentation performance is limited by the generated pseudo labels. In the future, we will explore the direction of semi-supervised learning if a separate set of pixel-wise labels is available for training.

## VII. Conclusion

In this paper, we propose the first weakly supervised learning framework for adipose tissue segmentation in human cardiac OCT images. We design a novel CAM-superpixel segmentation approach that converts the sparse CAM results into pseudo-pixel-wise labels for training. In addition, we also present and analyze the necessity and effectiveness of proposed steps and loss functions. Experimental results on the human cardiac dataset demonstrate that our model achieves comparable performance with models trained under full masks, showing the learning capability of our proposed model on image-level labels.

In the future, we will extend our work in the following aspects. First, we will evaluate the performance of our current work into catheter-based in-vivo OCT images or synthetic in-vivo OCT images generated by generative models and image kernels. Second, we will validate the effectiveness of our model on other OCT segmentation tasks to open up opportunities for broader applications.

## Figures and Tables

**TABLE I:** Clinical Characteristics of Heart Donors

Characteristic	Value
N	44
Demographic profile	
Age in years, median (average)	62 (62.2)
Female, n (%)	20 (45.5)

Medical history, n (%)	
Heart failure	10 (22.7)
Cardiomyopathy	8 (18.2)
Coronary artery disease	11 (25.0)
Myocardial infarction	10 (22.7)
Atrial fibrillation	3 (6.8)
Chronic obstructive pulmonary disease	16 (36.4)
Diabetes	17 (38.6)
Hypertension	27 (61.4)

Cause of death, n (%)	
Cardiac arrest	18 (40.9)
Cardiopulmonary arrest	2 (4.5)
Respiratory failure	5 (11.4)
Chronic obstructive pulmonary disease	1 (2.27)
Congestive heart failure	1 (2.27)
Others, cardiac related	11 (25.0)
Others, not cardiac related	6 (13.6)

**TABLE II:** Evaluation Metrics (%) of Adipose Tissue Seeds Before and After the Boundary Masking Algorithm

	Before	After
Accuracy	80.79 ± 1.15	83.91 ± 2.45
Precision	56.43 ± 11.95	75.90 ± 8.18

**TABLE III:** Evaluation Metrics (%) on Tissue Pseudo Labels Before and After the Spatial Regularisation on Adipose Samples

Method	True Positive Rate	False Positive Rate	Dice Coefficient
Superpixel	71.17 ± 6.36	10.03 ± 2.87	67.09 ± 2.96
Superpixel + Spatial regularisation	71.77 ± 6.72	8.33 ± 2.90	69.70 1 3.34

**TABLE IV:** Evaluation Metrics (%) on Pseudo Labels Generated from Different Models on Adipose Samples

Model	Precision	True Positive Rate (%)	False Positive Rate (%)	Dice Coefficient (%)
Deeper Model	0.93 ± 0.08	64.13 ± 10.76	5.38 ± 0.97	68.29 ± 7.18
Wider Model	0.92 ± 0.07	63.78 ± 3.98	**5.31 ± 1.50**	68.87 ± 1.59
Proposed	**0.93 ± 0.06**	**71.77 ± 6.72**	8.33 ± 2.90	**69.70 ± 3.34**

**TABLE V:** Evaluation Metrics (%) of Different Models on Whole Dataset

	Method	True Positive Rate	False Positive Rate	Dice Coefficient
Fully supervised baseline	U-Net	**82.32 ± 5.69**	**0.99 ± 0.90**	**81.57 ± 5.71**

Weakly supervised baseline	RRM	76.00 ± 16.36	5.44 ± 2.70	40.39 ± 9.01
	CycleGAN	55.19 ± 17.46	7.23 ± 6.44	64.14 ± 17.21

Proposed framework	U-Net	85.52 ± 5.44	1.35 ± 0.74	72.11 ± 10.12
	FCN	87.77 ± 1.01	1.49 ± 0.85	72.14 ± 8.94
	DeepLab	80.22 ± 4.18	1.45 ± 0.84	67.83 ± 1125

Ablation study	w/o Boundary masking	72.59 ± 8.46	1.37 ± 1.09	70.91 ± 16.95
	w/o Spatial regularization	77.84 1 5.67	1.21 ± 1.08	70.77 1 12.54
	Adipose seed loss + Dice loss	87.62 ± 8.79	2.24 ± 0.72	64.15 ± 9.82
	CE loss + Dice loss	74.04 ± 15.25	0.77 ± 0.30	74.99 ± 14.72

Except fully supervised method, the best and second best performance are marked in red and blue correspondingly.
